# Improved Health Status and Life Satisfaction among Older People following Self-Help Group Intervention in Jakarta

**DOI:** 10.1155/2017/3879067

**Published:** 2017-10-12

**Authors:** Junaiti Sahar, Ni Made Riasmini, Dwi Nurviyandari Kusumawati, Erna Erawati

**Affiliations:** ^1^Community Nursing Department, Nursing Faculty, Indonesia University, Depok, Indonesia; ^2^Nursing Doctorate Program, Nursing Faculty, Indonesia University, Depok, Indonesia; ^3^Politeknik Kesehatan Kementerian Kesehatan Jakarta III, Jawa Barat, Indonesia; ^4^Department of Psychiatric Nursing, Politeknik Kesehatan Kemenkes Semarang, Semarang, Indonesia

## Abstract

Although self-help group for older people gains the effectiveness, the effect of a nursing intervention using a self-help group model as a guideline for self-monitoring and intervention on the health status and life satisfaction among older people still remains. To determine the effectiveness of this nursing intervention, an experimental design using multistage sampling method was used for this study. The self-help intervention included a single 50–70-minute session once a week for 12 weeks done, using the Life Satisfaction Inventory-A (LSI-A) questionnaire, and SF-36 shows a significant difference. Self-help intervention could be implemented by nurses for older people in the community to improve health and well-being.

## 1. Introduction

The world population is rapidly increasing in age. As a developing part of the world, the population of Indonesia is 249 million with 8% of its population now aged over 60 years [1]. The average life expectancy in 1990 was 65.5 years, increasing to 70.6 years in 2009 [1, 2]. Most older people will eventually experience multiple health problems, such as coronary heart disease, stroke, arthritis, degenerative joint disease, and deteriorating mental health [2]. In 2006, Boonyakawee found that 87% of older people who have long-term disability become increasingly dependent on their family [3]. Older people with disabilities frequently have poor perceptions about their level of health [4, 5], and they become increasingly confined indoors [6].

The rapid increase in the number of older people in society and the aging process, together with increasing physical morbidity, will have an impact on society, families, and the provision of health care. Recently, the government of Indonesia has developed a program and policy to address these increasing problems due to the aging population by the implementation of individual empowerment using self-help groups to share experience and address problems in their lives, including health status and life satisfaction.

In elderly individuals, life satisfaction is a multidimensional issue that is influenced by objective and subjective characteristics, including cognitive function, emotional status, social support, physical condition, dependency, and sociodemographic variables [7]. Nurses can implement group interventions, such as healthy aging classes, to help individuals to cope more effectively with the life events of older age [8]. Through self-monitoring and intervention guidelines for older people in Indonesia, these programs have begun to be structured and managed. A new supportive environment may improve the ability of older people to do things that are important to them, despite significant limitations in their capacity. Self-help groups can be identified as an intervention to foster healthy aging by maximizing functional ability in two ways: by building and maintaining intrinsic capacity and by enabling individuals with the reduced functional capacity to do the things that are important to them. Building intrinsic capacity which refers to the composite of all the physical and mental capacities will develop and maintain the functional ability that enables well-being in older age [1].

Various studies proved that self-management or self-intervention for the patient with chronic illness can improve health and activity. Smeulders et al. [9] said that self-management program in 6 weeks and 2.5 hours per week gave a positive effect in managing the chronic disease. This is also supported by Whittle et al. [10]; self-management for hypertension significantly decreased blood pressure in the older adult. Panagioti et al. [11] explained that self-management can decrease the use of health service, although the effect was small. Other researches also proved that the self-management program gave a positive effect like improving patient activity so that it possible for the patient to do the self-care activity [12]. A study by Shin et al. [13] in South Korea in 2015 also proved that self-management empowerment program was effective for older people. The researches provide evidence that self-help group program followed with information about how to recognize various problems that may occur in older people can help to maintain health and life satisfaction. Haber and Lacy [14] through their study suggest that group intervention through socioeducation support improved health behavior and reduced stress. Dayton et al. [15] also found a group intervention for enhancing forgiveness associated with a short-term improvement in health status. The efficacy of self-help program with a formal self-help delivery model charged with reconstructing an older woman's social support system promotes comfort in sharing, group involvement, and social, intellectual, and emotional gain [16]. Moreover, socializing with others, they can decrease the dependency to the family. This thing is important to do by older people in developing countries like Indonesia where the service in the institution like a nursing home is not the first choice because the service provided focuses more on the social service than the nursing service. Older people often experience various health problems that affect the long-term condition, so that the effort to maintain their health through empowerment in health care is important to do. The aims of this study were to determine the effect of a nursing intervention, using a self-help group model as a guideline for self-monitoring and intervention on the health status and life satisfaction in elderly individuals.

## 2. Materials and Methods

An experimental design with a control group was applied. The population of this study was all older people in six administrative cities in Jakarta which consisted of Central Jakarta, West Jakarta, South Jakarta, East Jakarta, North Jakarta, and Kepulauan Seribu. We used sample size estimation with group comparison (two groups) in this study [17]. The sample size was based on a significance level of 5% (*Z*_1−*α*/2_ = 1.96) and power of 80% (*Z*_1−*β*_ = 0.84) to detect ≥20% difference in means. The final sample size was 94, but to anticipate participant drop out, we added 10%, which resulted in a final sample size of 105 older people for each group.

**Figure 1:**
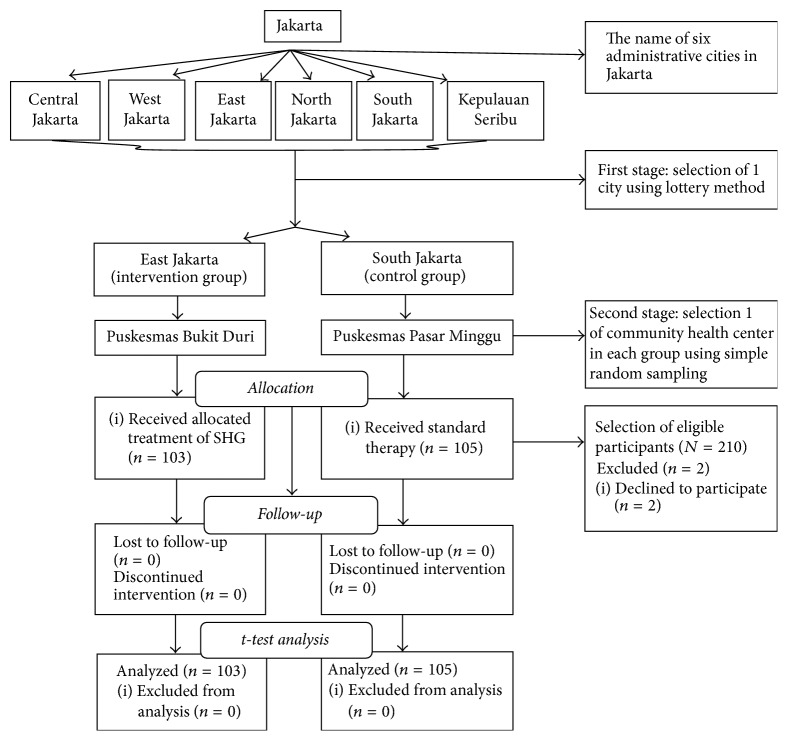
Consort flow chart. The intervention of SHG.

The elderly participants in this study were selected from 339 community health center services in Jakarta and represented 9.61% of the total population. A multistage sampling method was used in order to select the center for this study in Jakarta. In the first stage, we randomly selected from six administrative cities using lottery method, and the result was East Jakarta as an intervention group and South Jakarta as a control group. In the second stage, we randomly selected one community health center in each group and the results were Puskesmas Bukit Duri as an intervention group and Puskesmas Pasar Minggu District as a control group (see Figure 1).

To be eligible, the older people had to meet the following criteria: older people aged between 60 and 74 years; individuals living with their family; individuals not suffering from immobility; those individuals able to read, write, communicate well, and have willing to participate the trial. We selected eligible participants from intervention group (*N* = 105) and control group (*N* = 105).

At the beginning of study, two participants in the intervention group did not continue to participate in the study because they were visiting relatives in the village. Therefore, they were excluded in the data analysis. None of older people refrained to participate in this study.

Ethical approval for the study was obtained from the Ethics Committee of the Faculty of Nursing at Indonesia University, Jakarta. The study was conducted in accordance with the ethical standards laid down in the Declaration of Helsinki. All study participants gave full informed consent. All study participants were recruited through community health centers, and study participation was voluntary.

The psychiatric community nurses supported the trial but had no access to the trial data or the data analysis. The self-help group (SHG) was cofacilitated by a community psychiatric nurse with a focus on promoting client-client interaction. The IRB ethical approval number was 75/H2.F12.D/HKP.02.04/2013. The authors of this study were solely responsible for its design and conduct and for all study analysis.

*The Self-Help Group (SHG)*. A self-help group (SHG) was an intervention group and was an independent variable in this study, cofacilitated by a psychiatric community nurse working in the community health services centers, South Jakarta, Indonesia. At the beginning of the intervention, the psychiatric community nurses explain how to use the guidelines of self-monitoring and intervention as health promotion relates to self-management to minimize health problems and the impact of unhealthy behavior.

The twelfth session of the SHG was aimed at working specifically for the older people in the study during the 50–70-minute sessions that occurred once a week for 12 weeks. The first session was the initial stage of the study and included structuring, establishing group rules, ensuring participant confidentiality, and explaining the goals of the program and the goals of the group members. The second and third sessions of the SHG were the relational stages and included building relationships. The fourth to the eleventh sessions were the working stages: the fourth and fifth sessions included sharing thoughts, feelings, and behaviors; the sixth and seventh sessions included sharing experiences and awareness of the aging process and health problems; the eighth and ninth sessions included developing self-worth and positive practices; the tenth and eleventh sessions included follow-up and support; the twelfth session was the termination stage and included evaluation of all the previous sessions. Self-monitoring, feedback, and self-reinforcement techniques were used through the SHG sessions. During this period the control group received usual care through home visits. Psychiatric community nurses gave individual care based on the needs of the individuals for the control group.

### 2.1. Measures

The study included the use of three questionnaires: (1) questions on demographic information, (2) the individual health status, and (3) the individual life satisfaction. Sociodemography variables include age, gender, marriage status, race, education and work, the length of stay together within the family, the number of family members residing with the individual, and the family relationships. The family relationship describes how family members get connected with older people. Family members are available and responsible for caring to the elderly people.

One week before administering SHG for the intervention group and usual care for the control group, questionnaires were administered to both groups. Two weeks after the completion of SHG session, once again, both groups replied to the questionnaires. Then, a posttest analysis was applied.

#### 2.1.1. The Primary Outcome of Health Status

The short-form- (SF-) 36 questionnaire has been widely used for elderly. These scales measure health status physically and mentally. It contains eight items which is developed from Helgeson et al. [18] which include the perception of health, physical functioning, role performance due to physical changes, physical health, mental health, stability, emotional changes, and social functioning. The Indonesian of SF-36 version was administered in 5–10 minutes [19]. The instrument has been used in a previous study [18] in five districts in Jakarta. The reliability and validity of this instruments were high (ranging from 0.77 to 0.81). In this study, the instrument validity was ranged at 0.398. Internal consistency reliability of instruments was 0.747. The score range between 0 and 100, where 50 indicates norm and 100 indicates the best health state.

#### 2.1.2. The Secondary Outcome of Life Satisfaction

Life satisfaction analysis was based on the Life Satisfaction Inventory-A (LSI-A) [20]. The Indonesian version of the LSI-A consists of 20 items with the three-points scoring system. If the respondent checks off disagree response, the point is 0; unsure response, the point is 1; and agree response, the point is 2. The total scale score was based on the number of subject agreements with specific responses. In the previous research by Sahar et al. [21], the LSI-A was found to be reliable, producing a test-retest coefficient of 0.87 over two weeks, and an internal consistency of *α* = 0.747. In this study, the instrument validity was considered as acceptable at 0.4 or higher. Internal consistency reliability of instruments was consistently greater than 0.85. The possible range for LSI-A with one point given for each agreement is 0–40.

The questionnaires were administered to the intervention and the control group as a pre-test. Descriptive statistical methods were used for the data analysis. *T*-test analysis was conducted to identify the changes in health status and life satisfaction before and after the intervention. All statistical tests were conducted using SPSS software (Version 17, Chicago, IL, USA).

## 3. Results

The primary outcome of health status determined by the Short-Form-36 (SF-36) questionnaire showed that the mean score in the pre-test for the intervention group was 41.68 while the control group was 43.24. The secondary outcome life satisfaction using the Life Satisfaction Inventory-A (LSI-A) questionnaire showed that the mean score in the pre-test for the intervention group was 28.36 while the control group was 20.72. The intervention and the control groups did not differ significantly on outcome variable at baseline. Also, the length of stay together of older people with family using an independent *t*-test is shown in Table 1.

The sociodemographic variable analysis used the Chi-Square test. The percentage of age ranging from 65 to 69 years of the intervention group was 35% and the control group was 30.5%. Most respondents were female, most of them were still married, mostly having had a secondary school education, but they were mostly pensioners. Most respondents in the intervention group were Betawinese (42.7%), while in the control group, they were Javanese (42.9%). The number of family members caring for older people was predominantly less than three; the relationships with the older study participants and their family were mostly with a son or daughter, and the average length of stay together of older people with family was 31.04 years in the intervention group and 20.72 years in the control group. The intervention and the control groups did not differ significantly on any variable at baseline. These and other characteristics are summarized in Table 2.

### 3.1. Health Status

**Figure 2:**
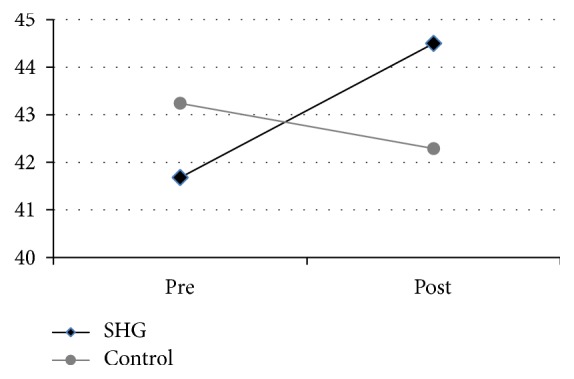
The pre-post difference in health status. Change scores (pre-post) for the SF-36. While the older people in the control group did not improve significantly, the SHG intervention group significantly gained performance at reassessment.

An independent *t*-test was used, with changes in the scores from the SF-36 serving as dependent variables.  Figure 2 shows that the average of the SF-36 score in the intervention group before the implementation of the self-help group (SHG) was 41.68 ± 5.27; after the implementation, the score increased to 44.50 ± 6.52. The average SF-36 score from the control group before the implementation of the SHG was 43.24 ± 8.60; after the implementation, the score decreased to 42.29 ± 5.15. There was a significant difference in the score of SF-36 for intervention group (*M* = 44.50, SD = 6.52) and for control group (*M* = 42.29, SD = 5.15), condition; *t*(1,41) = 3.54, *p* = 0.007. The pre-post-intervention difference in the SF-36 was significant in the intervention group (*t*-test; *p* < 0.05).

### 3.2. Life Satisfaction

**Figure 3:**
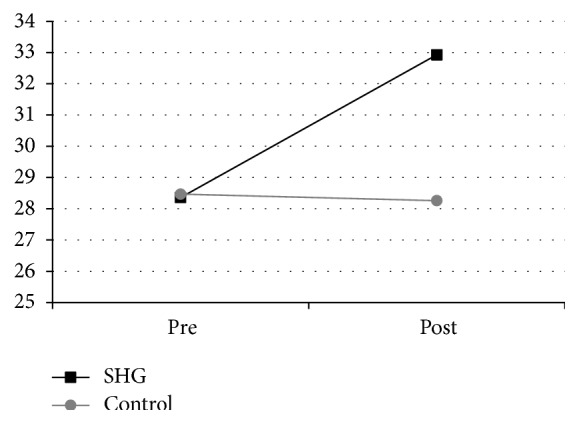
The pre-post difference in life satisfaction. Change scores (pre-post) for the LSI-A. While the older people in the control group did not improve significantly, the SHG intervention group significantly gained performance at reassessment.

Changes in life satisfaction were analyzed using an independent *t*-test.  Figure 3 shows that the average of LSI-A was 28.36 ± 4.16 for the SHG group and 28.47 ± 4.03 for the control group and increased to 32.93 ± 4.88 for the SHG group and decreased to 28.26 ± 4.89 for the control group. There was a significant difference in the score of SF-36 for the intervention group (*M* = 44.50, SD = 6.52) and for the control group (*M* = 42.29, SD = 5.15), condition; *t*(2.3) = 3.54, *p* = 0.007.

There was a significant difference in the score of LSI-A for intervention group (*M* = 32.93, SD = 4.88) and for control group (*M* = 28.26, SD = 4.89), condition; *t*(1.5) = 7.72, *p* ≤ 0.001. The pre-post-intervention difference in the LSI-A data was significant for the SHG group (*t*-test; *p* < 0.05) (see Figure 3).

## 4. Discussion

The results of this study have shown that the implementation of a self-help group (SHG) for older individuals can lead to a significant increase in health status and life satisfaction. One of the rationales behind the use of SHG intervention is that older individuals have a structured process that allows them to cope when they have outlived close friends and that helps them to begin socialization and make new friendships [22–24]. This study showed that the control group who did not receive self-help had reduced posttest scores, although they had usual care. The process of the SHG may be considered to be a part of nursing care in the community. Stanhope and Lancaster [25] have recently mentioned that nurses use their understanding of group principles to work with community groups to provide improved health changes. Self-help groups may also offer self-monitoring as a guide to the maintenance of health status and self-care. This finding would be consistent with the reports from Dale et al. [26] who through their research found that there was a significant correlation between the ability to provide self-care and the health status and quality of life. Therefore, through SHGs, older people may more easily develop relationships and become more aware of the importance of self-health management. This concept is supported by the findings of this study. Within the group, older people could reduce isolation and loneliness because they began to connect with others who have experience of aging [27]. The findings of this study are also consistent with the study results reported by Chao and colleagues [28] which showed that 1163 persons in a health management program based community showed improvement in physical health. Analysis from Whittle et al. [10] found that through peer-delivered self-management support, health conditions such as hypertension improved, with a significant decrease in systolic blood pressure. Anuruang et al. [29] reported that promoting effective self-care and self-management behaviors was critical to improving the clinical outcomes for chronic conditions.

In this study, group activity was done twice a week, with shared individual experiences that reinforced each other. This supports the result that the more the older people get connected with others, the better activation, assessment of care, and health behaviors and it could reduce psychosocial problems also [30]. Reeves et al. [31], through their findings, indicated that social involvement and groups could maintain personal self-management and physical and mental well-being. Reeves et al. also found that support work undertaken by personal networks expands in accordance with health needs, helping people to cope with their condition so that the use of SHGs could increase the physical and mental health of older individuals. Gentry et al. [32] also mentioned that self-management behaviors designed to promote patient autonomy contribute to positive health outcomes after therapeutic interventions. During the SHG study we have described, the intervention group responded that they knew how to overcome their problems. For example, when they woke up two or three times every night to urinate, they practiced Kegel exercises, as mentioned in self-help guidelines, resulting in fewer episodes of nocturia.

Despite the improvement of the result and new sight for community psychiatric nurse use SHG intervention for elderly, this study had several limitations. First, the study size was relatively small and analyzed individuals from a single, specific area of urban Indonesia. The study participants were taken from distinct areas that showed very different socioeconomic features. Most of the study participants were living with members of their family throughout the study; their selection, health factors, and quality of life would have been biased by their family support and family social interactions. Second, the reported finding only measures short-term outcomes using LSI-A and SF-36. Some authors noted motivation, social support, and social interaction during the process of SHG as the key for the improvement of health status and life satisfaction. This supports processes of change model of health behavior. But, older people may experience changes in motivation and intensity of self-management. It could impact the reported result. Previous studies focused on the effect of patient education and self-management support has found that short-term effects diminish significantly over time (at 6- and 12-month follow-up). Another study with social support in general as one of the outcomes was measured at 12-month follow-up for older women [33]. For future studies, long-term outcomes should have been used.

## 5. Conclusions

The current study results which suggest that the self-help intervention included a single 50–70-minute session once a week for 12 weeks could improve health status and life satisfaction. We recommend that self-help intervention may be implemented by nurses for older people in the community to improve health and well-being.

## Figures and Tables

**Table 1:** Statistics for performance variables among older people (*n* = 208).

Variable	Intervention (*n* = 103)		Control (*n* = 105)		*p*
	*M*	SD	*M*	SD	
SF-36	41.68	5.27	43.24	8.60	*p* = 0.7
LSI-A	28.36	4.16	28.47	4.03	*p* = 0.2
The length of stay together of older people with family	31.04	14.39	20.72	7.29	*p* = 0.1

*Note*. SF-36: short form functional health domain; LSI-A: Life Satisfaction Inventory-A; total score using independent *t*-test.

**Table 2:** The sociodemographic variables of older people at baseline (*n = *208).

Variable	Intervention (*N* = 103)		Control (*N* = 105)		*p*
	Frequency	Percentage	Frequency	Percentage	
*Age in years*					
≤64 years	44	42.7	36	34.3	*p* = 0.117
65–69 years	36	35.0	32	30.5	
≥70 years	23	22.3	37	35.2	

*Gender*					
Male	21	20.4	31	29.5	*p* = 0.173
Female	82	79.6	74	70.5	

*Marriage status*					
Married	69	67.0	58	55.2	*p* = 0.111
Widow/widower	34	33.0	47	44.8	

*Race*					
Betawi^a^	44	42.7	38	36.2	*p* = 0.780
Javanese^b^	29	28.2	45	42.9	
Sundanese^c^	30	29.1	22	21.0	

*Education*					
Not educated	2	1.9	10	9.5	*p* = 0.540
Secondary school	63	61.2	63	60.0	
Tertiary school	38	36.9	32	30.5	

*Working status*					
Employed	45	43.7	27	25.7	*p* = 0.100
Pensioners	58	56.3	78	74.3	

*Number of family members caring older people*					
<3	77	74.8	67	63.8	*p* = 0.119
≥3	26	25.2	38	36.2	

*Relationship with older people*					
Spouse	29	28.2	30	28.6	*p* = 0.640
Son/daughter	60	58.3	56	53.3	
Others	14	13.6	19	18.1	

^a^Betawi: An Indonesian ethnic group consisting of the descendants of the people living around Batavia (Jakarta); ^b^Sundanese: are an ethnic group native to the western part of the Indonesian island of Java; ^c^Javanese: are an ethnic group native to the Indonesian island of *Java*.
